# Complete Genome Sequences of the Escherichia coli Donor Strains ST18 and MFD*pir*

**DOI:** 10.1128/MRA.01014-20

**Published:** 2020-11-05

**Authors:** Simon A. Jackson, Bridget J. Fellows, Peter C. Fineran

**Affiliations:** a Department of Microbiology and Immunology, University of Otago, Dunedin, New Zealand; b Genetics Otago, University of Otago, Dunedin, New Zealand; c Bio-Protection Research Centre, University of Otago, Dunedin, New Zealand; Loyola University Chicago

## Abstract

Escherichia coli ST18 and MFD*pir* are donors commonly used to transfer *oriT*_RP4_-containing plasmids to diverse bacteria via conjugation. ST18 and MFD*pir* were constructed via multiple genetic manipulations involving several E. coli strains. Here, we used Illumina and Nanopore sequencing to determine the complete genomes of these widely used strains.

## ANNOUNCEMENT

A common laboratory method for transferring plasmids to diverse bacteria is via conjugation from an Escherichia coli donor strain. Several E. coli K-12 derivative strains have been engineered as donors, typically based on mobilization machinery derived from the broad-host-range plasmid RP4 (1–4). To facilitate efficient postmating donor counterselection, several auxotrophic E. coli donors, such as ST18, have been constructed (5). ST18 is a 5-aminolevulinic acid auxotroph derivative of S17-1 λ*pir* that was derived by deletion of *hemA* (5–8). During mating with S17-1 λ*pir* (by implication also with ST18), parts of the donor E. coli genome can inadvertently transfer to the recipient strain via Mu phage mobilization or high-frequency recombination (Hfr) transfer from an *oriT* located within the chromosome-integrated RP4 region (*oriT*_chrRP4_) (2, 3). To overcome these issues, a Mu-free donor strain, MFD*pir*, was constructed by removal of the Mu prophages and mutation of *oriT*_chrRP4_ to prevent Hfr transfer (2, 3). Additionally, MFD*pir* is a diaminopimelic acid auxotroph and *recA* deletion mutant (2). The construction of ST18 and MFD*pir* involved multiple genetic manipulations of several E. coli strains from the K-12 lineage (1–3, 5). To confirm the genotypes of these widely used donors, we determined the complete genome sequences of both strains. The ST18 strain reported here has undergone three transfers between research groups since the source laboratory (5). Therefore, there may be divergence between the isolate reported here and other laboratory strains in circulation. The MFD*pir* isolate reported here was obtained directly from the laboratory that constructed the strain (2).

Cultures of ST18 and MFD*pir* were grown in LB medium (10 g/liter Bacto tryptone, 5 g/liter yeast extract, and 5 g/liter NaCl) at 37°C for 20 h with shaking at 200 rpm and then pelleted by centrifugation. Genomic DNA was extracted using either a DNeasy blood and tissue kit (Qiagen) or a NucleoBond high-molecular-weight (HMW) DNA kit (Macherey-Nagel). Isolated genomic DNA was cleaned and concentrated using AMPure XP beads (Beckman Coulter). Short-read data (Illumina NextSeq custom library preparation, with 150-bp paired-end reads) for both strains and long-read data (Nanopore MinION platform, using the SQK-LSK109 library kit) for MFD*pir* were obtained through the Microbial Genome Sequencing Center (MiGS) (Pittsburgh, PA) (9). Nanopore data for ST18 were obtained using a MinION R9.4.1 flow cell (rapid barcoding kit SQK-RBK004), base called using Guppy (Oxford Nanopore Technologies), and demultiplexed using Deepbinner v0.2.0 (10). Raw Illumina reads (ST18, 1,508,686 reads; MFD*pir*, 2,736,618 reads) were trimmed and adapter sequences were removed using Trimmomatic v0.39 (11). The Nanopore Fast5 reads (ST18, 47,264 reads, with an *N*_50_ value of 10,262 bp; MFD*pir*, 275,897 reads, with an *N*_50_ value of 20,005 bp) were converted to Fastq files using Poretools v0.6.0 (12), and reads of <2,000 bp were discarded. The first 50 bp and last 20 bp of each Nanopore read were trimmed and reads with average quality scores of <9 were removed using NanoFilt v2.6.0 (13). The processed Illumina and Nanopore reads were used for hybrid genome assembly using Unicycler v0.4.9 with default parameters (14). Both ST18 and MFD*pir* genomes were resolved to single closed circular chromosomes (ST18, 4,825,686 bp, with 50.9% GC content; MFD*pir*, 4,686,434 bp, with 50.9% GC content). Coverage was determined by mapping the input reads to the final assemblies using Bowtie 2 (15) and minimap2 (16) for the Illumina and Nanopore reads, respectively. The coverages for ST18 were 74× (Illumina) and 46× (Nanopore) and for MFD*pir* were 143× (Illumina) and 507× (Nanopore).

### Data availability.

The complete genome sequences have been deposited in NCBI GenBank with accession numbers CP060709 (ST18) and CP060708 (MFD*pir*) and annotated with the NCBI Prokaryotic Genome Annotation Pipeline (PGAP) v4.12 (17, 18). Illumina and Nanopore sequence data have been deposited in the SRA under the accession number PRJNA658821.
